# Supplementary material for: CD3+ and CD8+ T-Cell-Based Immune Cell Score and PD-(L)1 Expression in Pulmonary Metastases of Microsatellite Stable Colorectal Cancer
Source: Cancers (Basel). 2022 Dec 29;15(1):206. doi: 10.3390/cancers15010206 (PMC9818882; doi:10.3390/cancers15010206)
Supplement: Supplementary file 1 [file cancers-15-00206-s001.zip › cancers-2098059-supplementary.pdf]

Supplementary material

Table S1. Spearman correlation table of the mean of CD3$^+$ and CD8$^+$ density and PD-1 and PD-L1 expression in pulmonary metastases and primary CRC

		met PD1 (TC)	met PD1 (IM)	met PDL1 (TCe)	met PDL1 (IC)
**Median of metastases T-cell density**	Correlation Coefficient	**0.631****	**0.697****	0.061	**0.405****
	Sig. (2-tailed)	<0.001	<0.001	0.627	0.001
	N	67	63	67	66
		**prim PD1 (TC)**	**prim PD1 (IM)**	**prim PDL1 (TCe)**	**prim PDL1 (IC)**
**Median of primary tumour T-cell density**	Correlation Coefficient	**0.449****	**0.595****	0.221	**0.361****
	Sig. (2-tailed)	<0.001	<0.001	0.096	0.005
	N	59	59	58	58

**. Correlation is significant at the 0.01 level (2-tailed).
*. Correlation is significant at the 0.05 level (2-tailed).

Table S2. Baseline characteristics of pulmonary metastasectomy patients according to ICS of primary colorectal tumour (n=61)

	ICS 0	ICS 1	ICS 2	*p*-value
	n (%)	n (%)	n (%)	
	13	35	14	
**Sex**				0.614
Female	5 (38.5%)	18 (52.9%)	8 (57.1%)	
Male	8 (61.5%)	16 (47.1%)	6 (42.9%)	
**Age (M;SD)**	67.2 (9.3)	66.9 (9.2)	71.7 (10.1)	0.368
**CCI**				0.481
1	9 (69.2%)	20 (58.8%)	8 (57.1%)	
2	4 (30.8%)	7 (20.6%)	3 (21.4%)	
≥3	0 (0.0%)	7 (20.6%)	3 (21.4%)	
**CRC stage**				0.223
1-2	3 (23.1%)	15 (44.1%)	2 (14.3%)	
3	7 (53.8%)	10 (29.4%)	8 (57.1%)	
4	3 (23.1%)	9 (26.5%)	4 (28.6%)	
**CRC location**				0.941
Colon	7 (53.8%)	17 (50.0%)	8 (57.1%)	
Rectum	6 (46.2%)	17 (50.0%)	6 (42.9%)	
**Neoadjuvant CRC**				0.876
No	8 (61.5%)	23 (67.6%)	9 (69.2%)	
Radiotherapy	1 (7.7%)	4 (11.8%)	2 (15.4%)	
Chemotherapy	1 (7.7%)	1 (2.9%)	1 (7.7%)	
Radio-chemotherapy	3 (23.1%)	6 (17.6%)	1 (7.7%)	
**DFI (d; MD; IQR)**	750 (67-1004)	364 (0-745)	193 (0-762)	0.222
**Synchronicity**				0.101
Synchronous	1 (7.7%)	4 (11.8%)	5 (35.7%)	
Metachronous	12 (92.3%)	30 (88.2%)	9 (64.3%)	
**No of pulmonary metastases**				0.209
1	6 (46.2%)	23 (67.6%)	11 (78.6%)	
≥1	7 (53.8%)	11 (32.4%)	3 (21.4%)	
**Size of largest PM (cm; MD; IQR)**	1.5 (1.2-2.2)	2.1 (1.2-3.5)	3.5 (2.5-3.6)	0.170
**Laterality of metastasis**				0.256
Unilateral	9 (69.2%)	29 (85.3%)	13 (92.9%)	
Bilateral	4 (30.8%)	5 (14.7%)	1 (7.1%)	
BRAF				>0.999

Wild-type	13 (100.0%)	31 (93.9%)	14 (100.0%)	
Mutant	0 (0.0%)	2 (6.1%)	0 (0.0%)	
**met PD-1 (TC)**				0.016
Low	5 (38.5%)	25 (73.5%)	5 (35.7%)	
High	8 (61.5%)	9 (26.5%)	9 (64.3%)	
**met PD-1 (IM)**				0.080
Low	8 (61.5%)	19 (63.3%)	4 (28.6%)	
High	5 (38.5%)	11 (36.7%)	10 (71.4%)	
**met PD-L1 (TCe)**				0.443
Low	13 (100.0%)	34 (100.0%)	13 (92.9%)	
High	0 (0.0%)	0 (0.0%)	1 (7.1%)	
**met PD-L1 (IC)**				0.082
Low	4 (30.8%)	5 (14.7%)	0 (0.0%)	
High	9 (69.2%)	29 (85.3%)	13 (100.0%)	
**prim PD-1 (TC)**				0.031*
Low	9 (69.2%)	19 (55.9%)	3 (21.4%)	
High	4 (30.8%)	15 (44.1%)	11 (78.6%)	
**prim PD-1 (IM)**				<0.001*
Low	12 (92.3%)	17 (51.5%)	2 (14.3%)	
High	1 (7.7%)	16 (48.5%)	12 (85.7%)	
**prim PD-L1 (TCe)**				0.702
Low	13 (100.0%)	32 (97.0%)	13 (92.9%)	
High	0 (0.0%)	1 (3.0%)	1 (7.1%)	
**prim PD-L1 (IC)**				0.002*
Low	12 (92.3%)	26 (78.8%)	5 (35.7%)	
High	1 (7.7%)	7 (21.2%)	9 (64.3%)	
**ICS of metastases**				0.074
Low	4 (30.8%)	5 (14.7%)	0 (0.0%)	
Intermediate	5 (38.5%)	23 (67.6%)	8 (57.1%)	
High	4 (30.8%)	6 (17.6%)	6 (42.9%)	

*Statistically significant at the 0.05 level

Table S3. Spearman correlation table of CD3+ and CD8+ densities between primary tumour and first pulmonary metastases.

		**met CD3$^+$ (TC)**	**met CD3$^+$ (IM)**	**met CD8$^+$ (TC)**	**met CD8$^+$ (IM)**
**met CD3$^+$ (TC)**	Correlation Coefficient	1.000	**0.670****	**0.856****	**0.654****
	Sig. (2-tailed)		<0.001	<0.001	<0.001
	N	67	65	67	65
**met CD3$^+$ (IM)**	Correlation Coefficient	**,670****	1,000	**,613****	**,798****
	Sig. (2-tailed)	<0.001		<0.001	<0.001
	N	65	65	65	65
**met CD8$^+$ (TC)**	Correlation Coefficient	**,856****	**,613****	1,000	**,774****
	Sig. (2-tailed)	<0.001	<0.001		<0.001
	N	67	65	67	65
**met CD8$^+$ (IM)**	Correlation Coefficient	**,654****	**,798****	**,774****	1,000
	Sig. (2-tailed)	<0.001	<0.001	<0.001	
	N	65	65	65	65
**prim CD3$^+$ (TC)**	Correlation Coefficient	0.004	0.189	0.103	**.290***
	Sig. (2-tailed)	0.977	0.153	0.428	0.026
	N	61	59	61	59
**prim CD3$^+$ (IM)**	Correlation Coefficient	0.061	0.209	0.162	0.192
	Sig. (2-tailed)	0.644	0.115	0.218	0.149
	N	60	58	60	58
**prim CD8$^+$ (TC)**	Correlation Coefficient	0.234	0.229	**0.259***	**0.350****
	Sig. (2-tailed)	0.070	0.081	0.044	0.007
	N	61	59	61	59
**prim CD8$^+$ (IM)**	Correlation Coefficient	**0.354****	**0.350****	**0.362****	**0.406****
	Sig. (2-tailed)	0.006	0.008	0.005	0.002

	N	59	57	59	57

**. Correlation is significant at the 0.01 level (2-tailed).
*. Correlation is significant at the 0.05 level (2-tailed).

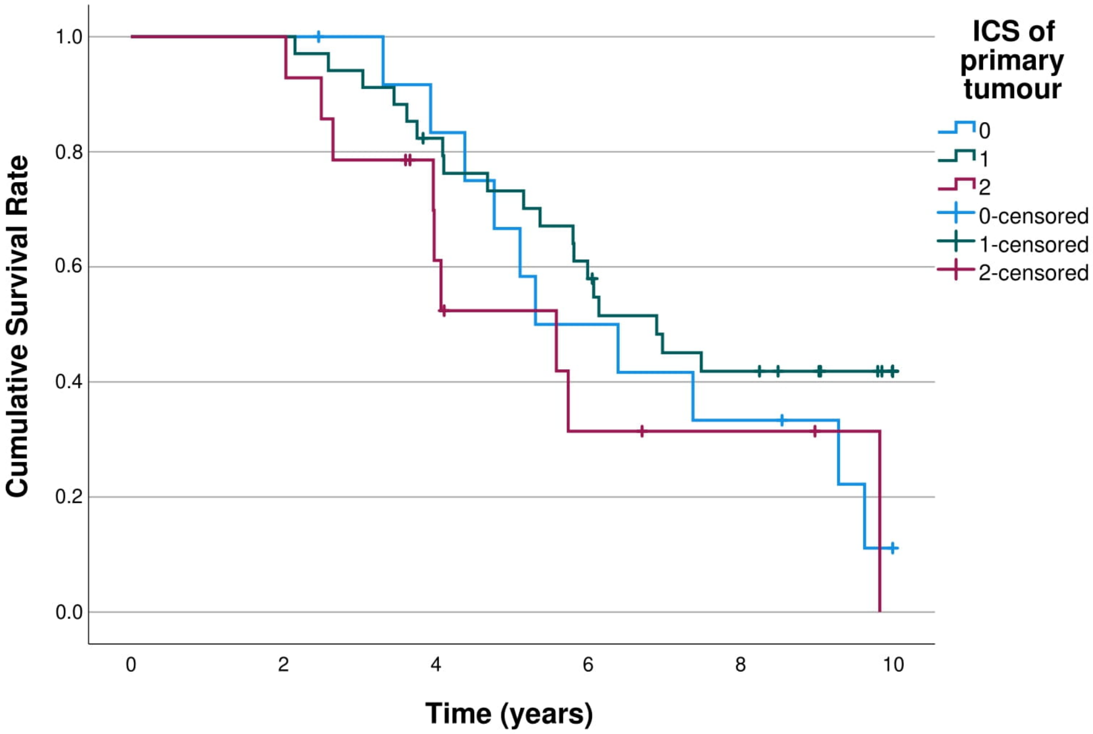

Figure S1. K-M curves of 10-year survival stratified by the ICS of the primary tumour. Log rank *p*=0.152.

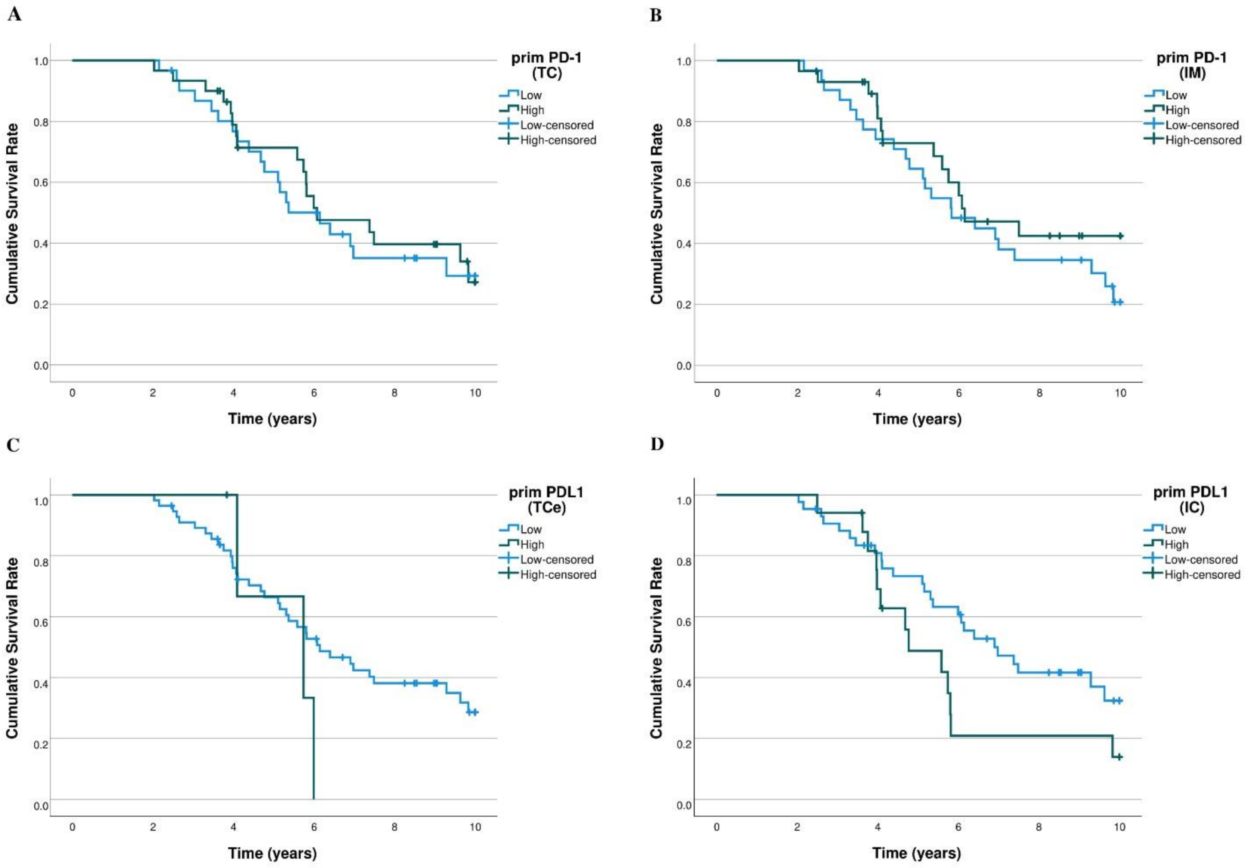

Figure S2. K-M 10-year overall survival curves of PD-1/PD-L1 expression of the primary tumour. **(A)** PD-1 expression (low vs. high) in the tumour centre (p=0.726). **(B)** PD-1 expression (low vs. high) in the invasive margin (p=0.328). **(C)** PD-L1 expression (low vs. high) in the tumour cells (p=0.504). **(D)** PD-L1 expression (low vs. high) in the tumour infiltrating immune cells (p=0.086).

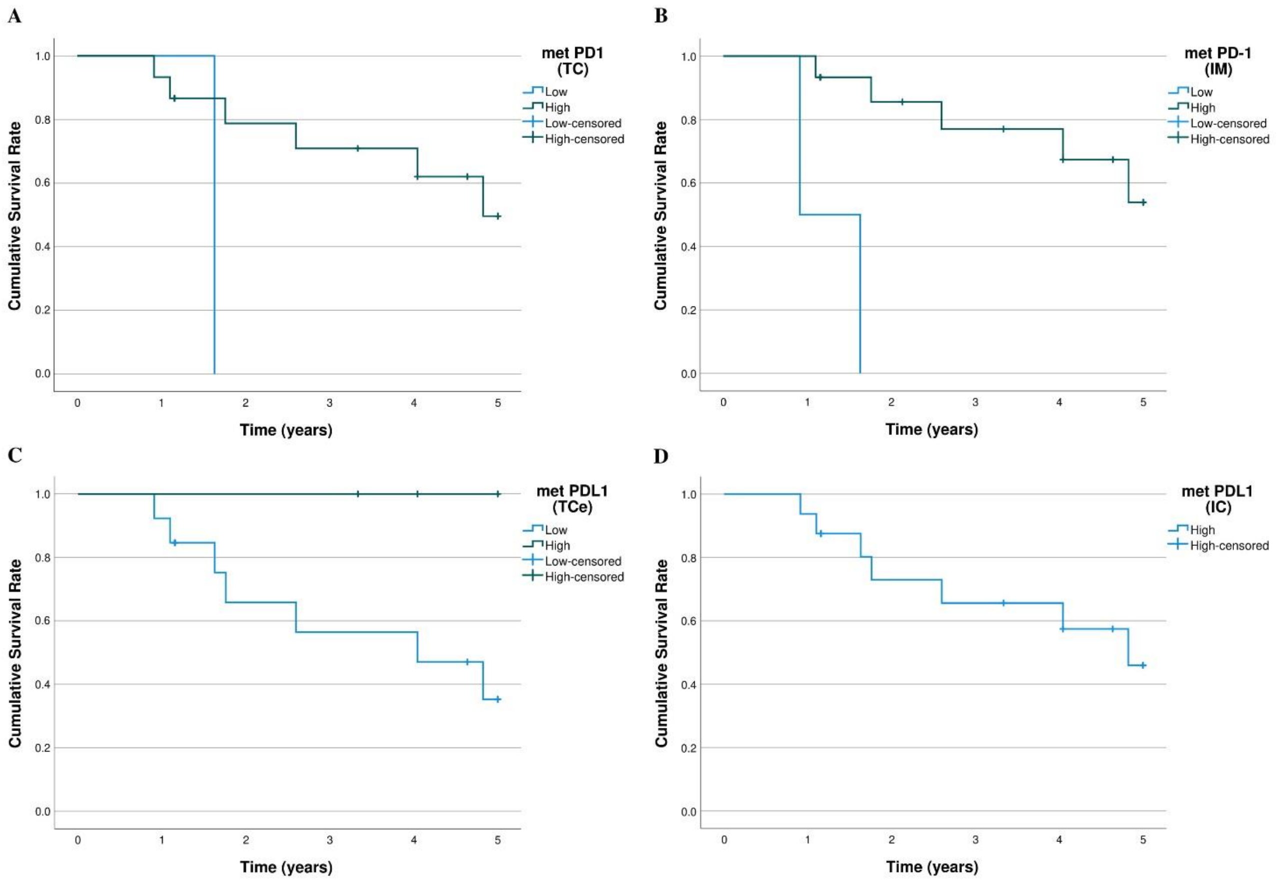

Figure S3. The K-M curves of 5-year overall survival of pulmonary metastasectomy in ICS-high pulmonary metastases (n=17) stratified by (**A)** PD-1 in the tumour centre (p=0.076), (**B**) PD-1 in the invasive margin (p<0.001), (**C**) PD-L1 in the tumour cells (p=0.138) and, (**D**) PD-L1 in the immune cells. Log rank tests were applied.

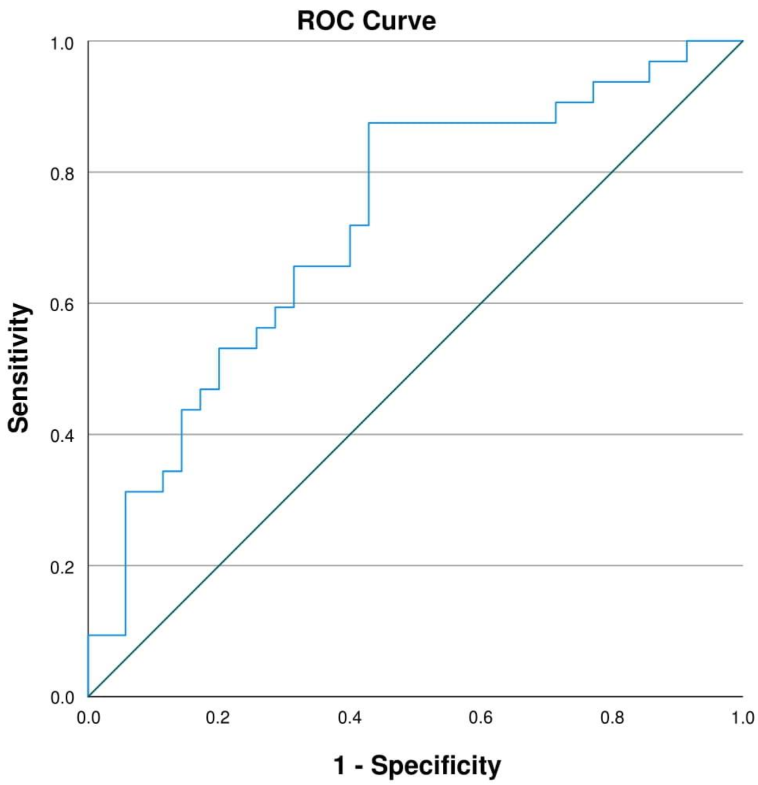

Figure S4. Receiver operating characteristics (ROC) curve of the mean percentile score of the immune cell densities in the invasive margin and tumour centre of the pulmonary metastases.

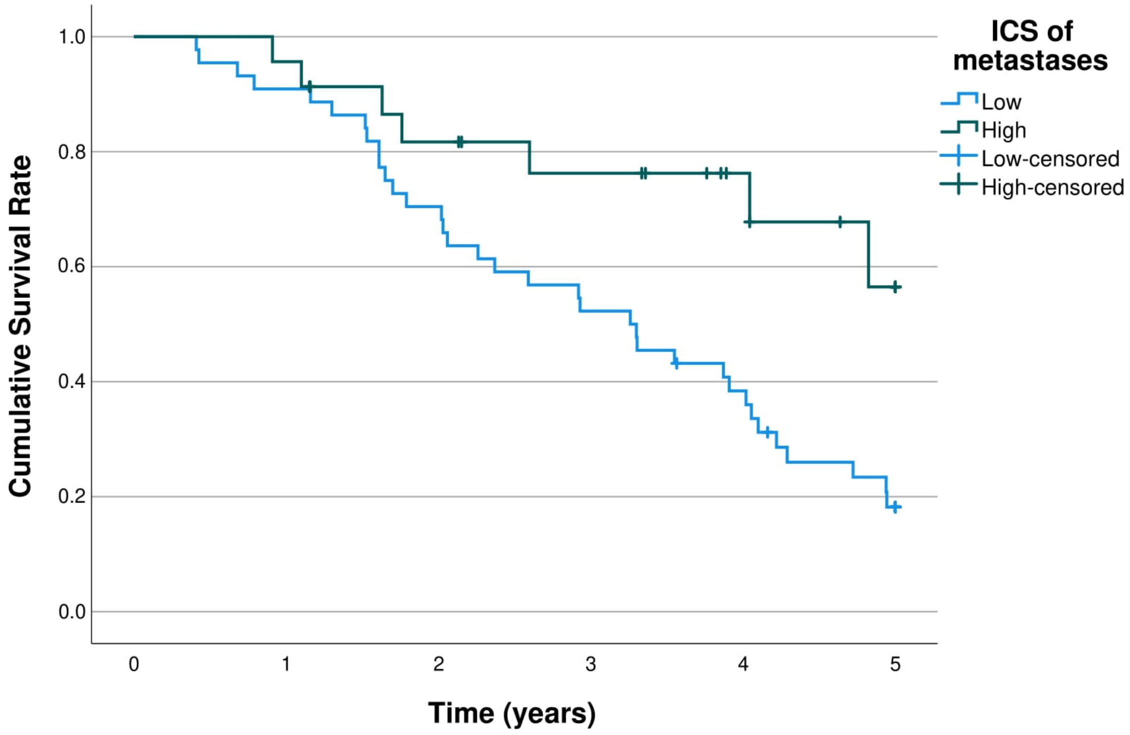

Figure S5. The K-M curves of 5-year overall survival stratified by the ICS of pulmonary metastases according to the cut-off value (65%) selected from ROC-curve (n=67). Log rank p=0.009.
